# Supplementary material for: Comparative Evaluation of the Antimicrobial and Mucus Induction Properties of Selected Bacillus Strains against Enterotoxigenic Escherichia coli
Source: Antibiotics (Basel). 2020 Nov 27;9(12):849. doi: 10.3390/antibiotics9120849 (PMC7760508; doi:10.3390/antibiotics9120849)
Supplement: Supplementary file 1 [file antibiotics-09-00849-s001.pdf]

antibiotics

Communication

Comparative Evaluation of the Antimicrobial and Mucus Induction Properties of Selected *Bacillus* Strains Against Enterotoxigenic *Escherichia coli*

Natalia Bravo Santano [1], Erik Juncker Boll [2], Lena Catrine Capern [2], Tomasz Maciej Cieplak [2], Enver Keleszade [1], Michal Letek [3,†] and Adele Costabile [1,*,†]

[1] Department of Life Sciences, University of Roehampton, London, UK; nbravosantano@gmail.com (N.B.S.); keleszae@roehampton.ac.uk (E.K.)
[2] Animal Health Innovation, Chr. Hansen A/S, Hørsholm, Denmark; dkerbo@chr-hansen.com (E.J.B.); dkleca@chr-hansen.com (L.C.C.); dktoci@chr-hansen.com (T.M.C.)
[3] Departamento de Biología Molecular, Facultad de Ciencias Biológicas y Ambientales, Universidad de León, León, Spain; michal.letek@unileon.es
* Correspondence: adele.costabile@roehampton.ac.uk, Tel.: +44-(0)-20-8392-3571
† These authors share co-senior authorship.

Received: 8 November 2020; Accepted: 25 November 2020; Published: date

Supplementary materials

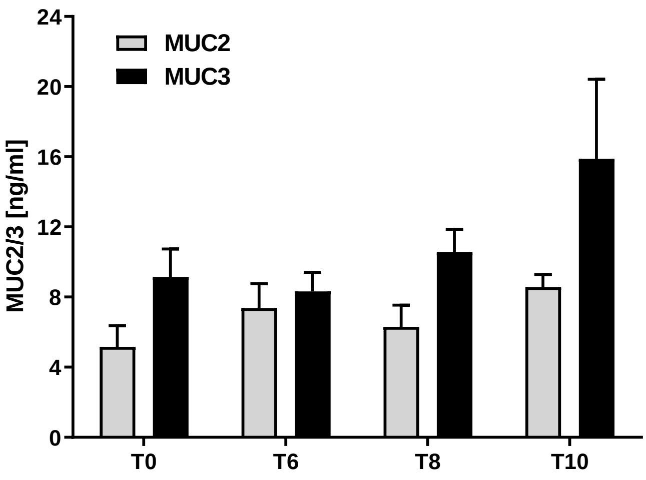

Figure S1. Quantification of MUC2 and MUC3 in HT29-16E cells after treatment with *Bacillus subtilis* strain CHCC 15541. HT29-16E cells were incubated with CHCC 15541 for 6, 8, and 10 hours, and concentrations of both mucin 2 (MUC2) and mucin 3 (MUC3) were quantified by ELISA at these time points. Data are expressed as Mean ± SEM of one experiment performed in duplicates.
